# Who’s in the House: Staffing in Long-Term Care Homes During the COVID-19 Pandemic

**DOI:** 10.1093/geroni/igab046.556

**Published:** 2021-12-17

**Authors:** Shirin Vellani, Charlene Chu, Annica Backman, Astrid Escrig-Pinol, José Tomás Mateos, Franziska Zúñiga, Karen Spilsbury, Katherine McGilton

**Affiliations:** 1 Toronto Rehabilitation Institute-University Health Network, Toronto, Ontario, Canada; 2 University of Toronto, Toronto, Ontario, Canada; 3 Umea University, Umea, Vasterbottens Lan, Sweden; 4 The Mar Nursing School, Universitat Pompeu Fabra, Barcelona, Catalonia, Spain; 5 University of Lleida, Lleida, Catalonia, Spain; 6 University of Basel, Basel, Basel-Stadt, Switzerland; 7 University of Leeds, University of Leeds, Leeds, England, United Kingdom; 8 KITE-Toronto Rehabilitation, University Health Network, Toronto, Ontario, Canada

## Abstract

There is an absence of high-quality workforce data that could be used globally for comparative research on workforce planning in the residential long-term care (LTC) sector. We know that older adults residing in the LTC settings have multimorbidities resulting in complex care needs, yet the workforce is insufficiently able to meet their needs. A further reduction in LTC workforce was noted during the COVID-19 pandemic which increased the risk of adverse outcomes for residents. Survey results focused on the workforce in LTC homes collected from several countries during the current pandemic, highlighted that several members of the workforce were either absent or worked virtually (e.g., physicians, social workers). A better understanding of who is/or should be in the house to meet the needs of residents during or after future pandemics requires a workforce data system that routinely collects this information to ensure best quality outcomes for residents and their carers.

